# Can Binocular Alignment Distinguish Hypertropia in Sagging Eye Syndrome From Superior Oblique Palsy?

**DOI:** 10.1167/iovs.63.10.13

**Published:** 2022-09-22

**Authors:** Qi Wei, Robert A. Clark, Joseph L. Demer

**Affiliations:** 1 Department of Bioengineering, George Mason University, Fairfax, Virginia, United States; 2 Department of Ophthalmology, University of California, Los Angeles, California, United States; 3 UCLA Stein Eye Institute, University of California, Los Angeles, California, United States; 4 Bioengineering Department, University of California, Los Angeles, California, United States; 5 Department of Neurology, University of California, Los Angeles, California, United States

**Keywords:** comitance, hypertropia, machine learning, magnetic resonance imaging, sagging eye syndrome, superior oblique palsy

## Abstract

**Purpose:**

Although the three-step test (3ST) is typically used to diagnose superior oblique palsy (SOP), sagging eye syndrome (SES) has clinical similarities. We sought to determine if alignment measurements can distinguish unilateral SOP from hypertropia in SES.

**Methods:**

We studied hypertropic subjects who underwent surface-coil magnetic resonance imaging (MRI) demonstrating either SO cross-section reduction indicative of congenital or acquired palsy (SOP group) or lateral rectus muscle sag (SES group). Alignment was measured by Hess screen and prism-cover testing. Multiple supervised machine learning methods were employed to evaluate diagnostic accuracy. Rectus pulley coordinates were determined in SES cases fulfilling the 3ST.

**Results:**

Twenty-three subjects had unilateral SOP manifested by SO atrophy. Eighteen others had normal SO size but MRI findings of SES. Maximum cross-section of the palsied SO was much smaller than contralaterally and in SES (*P* < 2 × 10^−5^). Inferior oblique cross-sections were similar in SOP and SES. In both SOP and SES, hypertropia increased in contralateral and decreased in ipsilateral gaze and was greater in ipsilateral than contralateral head tilt. In SES, nine subjects (50%) fulfilled the 3ST and had greater infraplacement of the lateral than medial rectus pulleys in the hypotropic orbit. Supervised machine learning of alignment data distinguished the diagnoses with areas under the receiver operating curves up to 0.93, representing excellent yet imperfect differential diagnosis.

**Conclusions:**

Because the 3ST is often positive in SES, clinical alignment patterns may confound SES with unilateral SOP, particularly acquired SOP. Machine learning substantially but imperfectly improves classification accuracy.

Although published papers^1^^–^^4^ and textbooks^5^^,^^6^ dealing with vertical strabismus typically assert that superior oblique palsy (SOP) is the most common cause of vertical diplopia or the most common paralysis of a single cyclovertical muscle,^7^ the basis of this time-worn belief may be questionable, insofar as the diagnosis of SOP has typically been the default presumption in cyclovertical strabismus unless an alternative diagnosis was obvious. Thus the diagnosis of “superior oblique palsy” has often been broadly applied to a wide range of cases of hypertropia (HT) for which no other classical mechanistic diagnosis has been established.^8^ Half of the cases captured in a strabismus clinic by such an approach are probably not related to a trochlear nerve or SO muscle lesion at all.^9^ For example, computational simulations indicate that displacement of the rectus muscle pulleys can mimic the horizontal and vertical patterns of incomitance caused by unilateral SO weakness.^10^

It has been widely assumed that the response of HT in SOP to head tilting provides diagnostic specificity.^7^ The Parks–Bielschowsky three-step test (3ST) has long been the workhorse for diagnosing cyclovertical strabismus.^6^ A positive 3ST in unilateral SOP consists of ipsilesional central gaze HT that is greater in contralesional than ipsilesional gaze and greater in ipsilesional than contralesional head tilt.^3^^,^^11^^,^^12^ The presumed physiological basis of the 3ST is that unopposed activity of the antagonist of the palsied SO, the inferior oblique (IO) muscle, increases the HT in contralateral gaze.^13^ However, computational simulations indicate that SO weakness alone cannot account for the typical magnitude of HT in SOP.^14^^,^^15^ Upshoot and downshoot in adduction, along with horizontally incomitant hypertropia, are inducible in normal humans^16^ and monkeys^17^ by only 3 to 7 days of monocular occlusion. Measurement during strabismus surgery has occasionally demonstrated SO contractile force generation despite motility consistent with palsy.^18^ The effect of head tilt is supposed to result from a deficit of incycloduction of the palsied SO during ocular counter-rolling,^12^ a deficit theorized to be replaced by ipsilateral superior rectus contraction that increases HT during ipsilateral head tilt.^19^ Based on this logic, when all 3ST steps are positive, many clinicians feel confident of SO weakness, notwithstanding widely variable HT incomitance that has been attributed to secondary changes such as IO overaction and superior rectus contracture.^1^^,^^20^ Thus, the 3ST, upon which most authors^21^ have conventionally relied despite clinically known errors,^22^ has been shown to be only 70% sensitive^23^ and 50% specific for actual SOP.^9^

The situation has become even more ambiguous since recent recognition of the sagging eye syndrome (SES) as a common cause of cyclovertical strabismus in people over 40 years old.^24^^–^^30^ Degeneration of the connective tissues in the orbital pulley system permits the lateral rectus (LR) muscle path to “sag” inferiorly in the orbit, converting some of its abducting force to infraduction. When this sag is bilaterally asymmetrical, the eye with greater LR sag becomes hypotropic, usually with greater excyclotropia than its hypertropic fellow.^24^ About two-thirds of all acquired diplopia in an adult population in Los Angeles is due to cyclovertical strabismus, of which SES causes twice as many cases as does SOP.^31^ It therefore is important to determine if the 3ST can distinguish SOP from SES in people who have acquired HT.

The present report sought to avoid confounding by considering only cases where magnetic resonance imaging (MRI) confirmed the distinct anatomical features of each diagnostic category. Atrophy of the ipsilateral SO muscle belly was considered to be a reliable and objective feature confirming unilateral SOP. We have previously demonstrated that experimental neurectomy of the subarachnoid trochlear nerve in monkey produces atrophy of the SO belly within 5 weeks, demonstrable both histologically and by MRI,^32^ and that humans with unilateral SOP exhibit MRI evidence of reduced SO size and loss of contractile thickening in infraduction.^33^ This study therefore included only cases of SOP in which quasi-coronal plane MRI demonstrated atrophic reduction in ipsilateral SO muscle cross-section. Also excluded on clinical grounds were neuromuscular disorders such as ocular myasthenia gravis and chronic progressive external ophthalmoplegia, where SO function might be dissociated from structure.

Although typical adnexal features of SES, such as blepharoptosis, superior sulcus defect, and eye laxity, are readily recognizable clinically,^34^ MRI findings are objectively diagnostic. These findings on quasi-coronal MRI include inferior displacement of the LR muscle path; thinning, elongation, or rupture of the LR–superior rectus (SR) band ligament; and temporal tilting of the superior relative to inferior long axis of the LR cross-section.^24^ This study therefore included only cases of SES in which quasi-coronal plane MRI demonstrated these findings and did not demonstrate atrophic reduction in SO muscle cross-section.

In some circumstances it might be clinically harmless to confuse SES with acquired SOP because of the typically benign course and associations of both conditions, as well as the similarity of therapies likely to be prescribed to relieve the associated diplopia. But, a dilemma may arise when a patient presents with acute onset of vertical binocular diplopia due to incomitant HT in a situation where acute neurological pathology is plausible. In such a situation, it may be important to distinguish HT due to the benign condition SES from potentially more ominous SOP that might be caused by an acute neurological lesion. The present study therefore ascertained the prevalence of positivity of the 3ST, and of each of its components, in groups of patients rigorously confirmed by MRI to have either SES or SOP. Machine learning (ML) can sometimes identify patterns in data that may have escaped human recognition.^35^ We therefore employed ML analysis of prism-cover alignment data, along with Hess screen tests, to discover the maximum differential diagnostic information that could theoretically be gleaned to distinguish HT due to SES from unilateral SOP.

## Methods

Institutional Review Board approval was obtained for a prospective study of strabismus conducted for more than the past 25 years. As described elsewhere,^36^ this study has recruited strabismic patients for detailed testing of eye movements, binocular alignment, and surface coil MRI of the orbits. Approximately 750 such strabismic volunteers contributed during the decades of the study and are cataloged by diagnosis. Recruitment was not designed to evenly sample all potential causes of strabismus. We considered data from all participants who had undergone digitally stored, quasi-coronal plane MRI of each orbit imaged separately using surface coils during target fixation by the scanned eye. Of these, we included all cases with high-quality MRI documenting unilateral SO atrophy who had undergone complete analysis of binocular alignment by prism/cover and Hess screen testing in all diagnostic gaze positions; had not undergone any prior strabismus, retinal, glaucoma, or orbital surgery; and were free of any neuromuscular disease, myopathy, or other potentially confounding condition. These cases were considered to have SOP. Cases were also excluded if there was any other cranial nerve palsy, significant horizontal strabismus not typically associated with SOP, or neuromuscular disorder such as ocular myasthenia gravis or chronic progressive external ophthalmoplegia.

Also, from the 750 total strabismus cases, we selected all cases of SES manifested by degeneration of the LR–SR band ligament with inferior displacement of the LR muscle path. From these cases, we selected all cases where the strabismus was predominantly cyclovertical, after excluding all cases with significant age-related distance esotropia that sometimes also includes a slight HT. These patients comprised the SES group.

High-resolution MRI of each orbit separately was performed as described elsewhere.^33^^,^^36^^–^^39^ Imaging with T1- or T2-weighted sequences was performed in 2-mm-thick, contiguous planes with an 80-mm square field of view and 256 × 256 matrix, yielding 312-µm in-plane resolution. For the SO, quasi-coronal planes were employed perpendicular to the long axis of the orbit; for the IO, quasi-sagittal planes were employed parallel to the long axis. Patients monocularly fixated a centered target with the scanned eye. Imaging was performed supine in most patients, but in both right and left lateral decubitus in a few with SOP. Because decubitus positioning causes slight SO contraction during ipsilateral and slight relaxation during contralateral head tilting,^40^ the data were averaged for the two postures. However, counter-rolling has an effect negligible in relation to SOP.^10^^,^^33^^,^^41^^–^^43^

All subjects included in this study had presented to the senior author for strabismus evaluation. Facial asymmetry was not considered a reliable diagnostic criterion.^44^ All patients underwent complete ophthalmological history and examination, including evaluation of ocular versions, and measurement of binocular alignment using prism-cover testing in both horizontal and vertical secondary gaze positions and with lateral head tilts at 4-m distance. Prism-cover measurements reported here represent maximum values revealed through dissociation through alternate covering of each eye. Torsion was measured as the difference in angular orientation of double Maddox rods in central gaze.

Subjects with SES were divided for analysis into groups according to how many components of the 3ST were fulfilled by their strabismus patterns. By definition, all included subjects with SES had HT at least in central gaze, but this was the only step fulfilled in some of them. Subjects with SES fulfilling only two steps also had either hypertropia greater in the contralateral than in the ipsilateral horizontal version or greater head tilt greater to the ipsilateral than contralateral shoulder. Consistent with prior study of SOP, an alignment difference of as little as 1∆ was considered a significant variation in HT in interpretation of the 3ST.^23^

Hess screen testing was performed using the Clement–Clark red light-emitting diode (LED) tangent screen array at 50 cm,^45^ with a green streak laser pointer directed by the patient. Dichoptic dissociation was achieved by having patients view, through a red filter, a closely spaced row of three red LED lights sequentially illuminated in each of 21 target positions as the patient directed a green laser streak to superimpose with the targets. The eye viewing through the red filter was designated as the fixing eye, and the eye viewing through the green filter was designated as the following eye. Sequentially illuminated targets were in central gaze, in ±15° and ±30° secondary horizontal and vertical gaze positions, and in tertiary combinations excluding corners. Left–right reversal of the viewing goggles permitted each eye to serve as fixing eye, doubling the test positions to 42. The horizontal and vertical alignment responses at each target position were marked on the published Clement–Clark Hess screen form. Hess screen charts were digitally scanned, and the horizontal and vertical deviations at each target position were quantified by a custom-developed algorithm written in MATLAB (MathWorks, Natick, MA). This converted the original Hess screen charts into a fully digital format for analysis. The Clement–Clark Hess screen does not reliably indicate ocular torsion.

The MRIs were evaluated digitally using Fiji.^46^ Cross-sectional areas were automatically determined after manual outlining using a digital cursor, and the maximum value was recorded for each orbit. Measured values were not corrected for SO path obliquity relative to the imaging plane. IO size was evaluated in the quasi-sagittal image plane closest to the midpoint of the inferior rectus muscle, as this is the most reliable region for this evaluation.^47^ Rectus pulley locations were determined only in subjects with SES who fulfilled the complete 3ST, using the method and coordinate system we have previously published.^10^^,^^24^ Statistical analysis was performed conventionally using analysis of variance (ANOVA) and paired and unpaired two-sided Student's *t*-tests.

**Figure 1.**
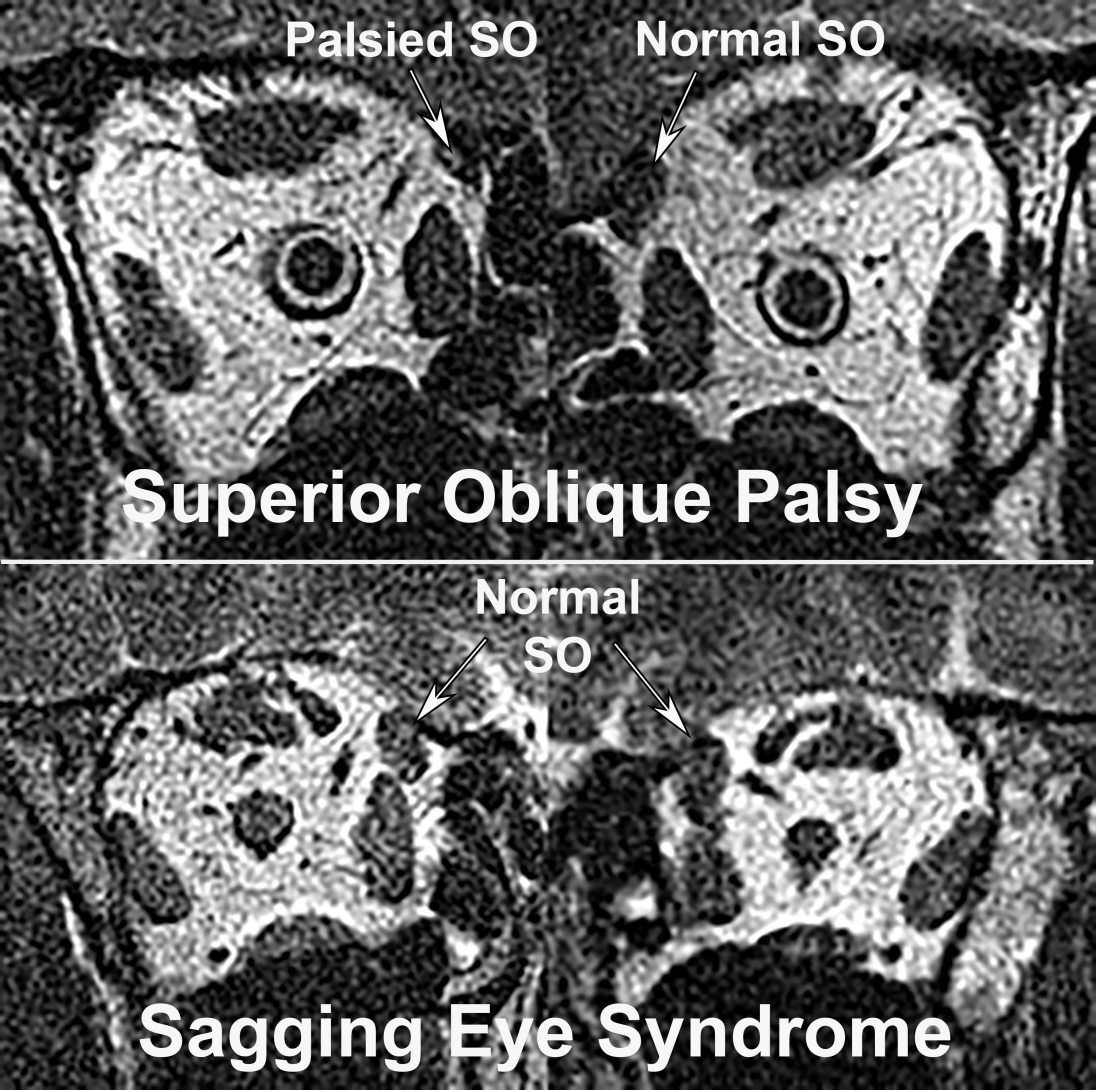
Quasi-coronal MRI in mid-orbital MRI planes in two subjects with right hypertropia due to (**A**) right SOP, demonstrating reduced right SO cross-section in central gaze, and (**B**) SES with normal SO cross-sections. See Figure 2 for anterior images. LPS, levator palpebrae superioris muscle; MR, medial rectus muscle.

The presence of unilateral SOP was confirmed by demonstration of unilateral atrophy of the SO muscle evidenced by reduction in maximum cross-sectional area in quasi-coronal MRI planes. This atrophy was confirmed by examination of additional image planes anterior and posterior to the plane of maximum cross-section. Figure 1A illustrates the MRI appearance of this unilateral atrophy for a case of acquired right SOP. The unilateral SO atrophy may be contrasted with the similar, normal SO cross-sections for the cases of SES in Figure 1B.

**Figure 2.**
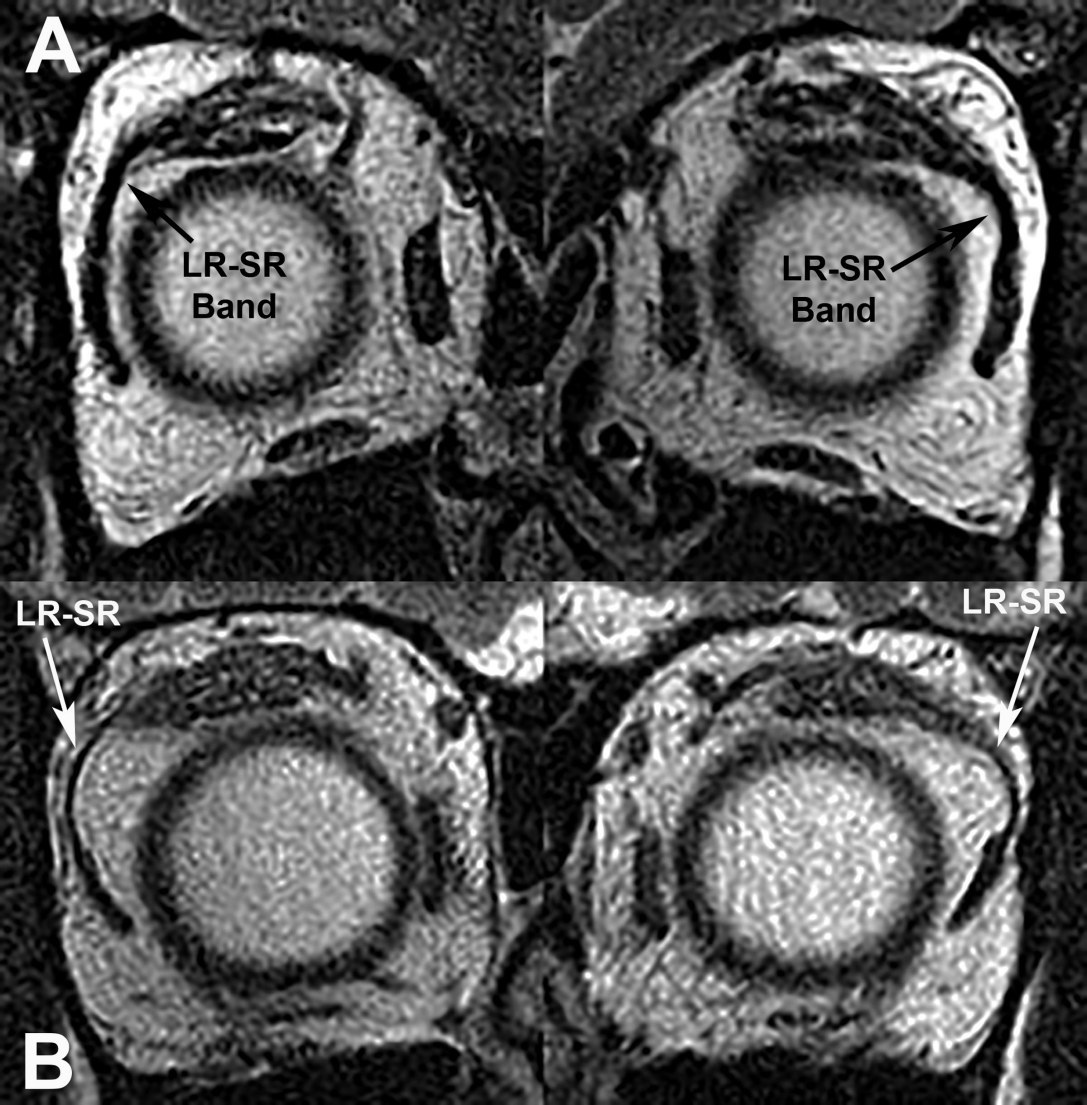
Quasi-coronal MRI in anterior planes in the subjects from Figure 1 with right hypertropia due to (**A**) SOP, demonstrating bilaterally robust LR–SR band ligaments and LR muscles in similar vertical position to the MR muscles, and (**B**) SES with attenuated, bulging LR–SR bands and inferiorly displaced, obliquely oriented LR muscles.

The existence of SES was confirmed in more anterior MRI planes by the demonstration of LR–SR band degeneration and inferior displacement of the LR path. These changes were required to be absent in cases diagnosed with SOP (Fig. 2A) but were obvious in cases diagnosed with SES (Fig. 2B). No case was included in which SO atrophy coexisted with features of SES.

For all cases, the IO muscle cross-section was analyzed in the quasi-sagittal image plane closest to the mid-point of the inferior rectus muscle, previously reported to be the best indicator of IO size.^48^

### Analysis by Machine Learning

Several supervised ML algorithms were used to examine their efficacy in distinguishing SES from SOP based on alignment measurements. The following classification methods (classifiers) implemented in the MATLAB Classification Learner app were used: decision tree, K-nearest neighbor, support vector machine, naïve Bayes, and ensemble classifier. Hyperparameters of these methods were optimized by the MATLAB optimizer. In order to prevent overfitting the data and to confirm generalizability of the ML models to independent data, we assessed classifier performance by fivefold cross-validation that trains and tests the ML models using different partitions of the data.

## Results

### Subjects

Of the 23 subjects with unilateral SO atrophy, nine cases (mean age, 38 ± 16 years) either gave unequivocal history of congenital onset of strabismus and head tilt or exhibited the ability to intermittently fuse HT of at least 20∆ for a distance target, constituting 10-times maximum normal vertical fusional vergence.^49^ These nine cases were considered to have congenital SOP. Fourteen cases (mean age, 52 ± 14 years) had acquired vertical diplopia symptomatic for 7 ± 6 years; these subjects were considered to have acquired SOP. Some alignment data on cases with SOP have been published previously in a report of vertical incomitance of HT,^36^ but that report did not include 3ST data.

Also identified were 63 cases of SES, from whom were selected all 18 cases where the strabismus was predominantly cyclovertical, excluding significant age-related distance esotropia with small HT. Subjects with SES averaged 60 ± 11 years of age and had experienced diplopia for 2.5 ± 4.2 years. These 18 subjects comprised the SES group.

### Confirmation of SOP

**Figure 3.**
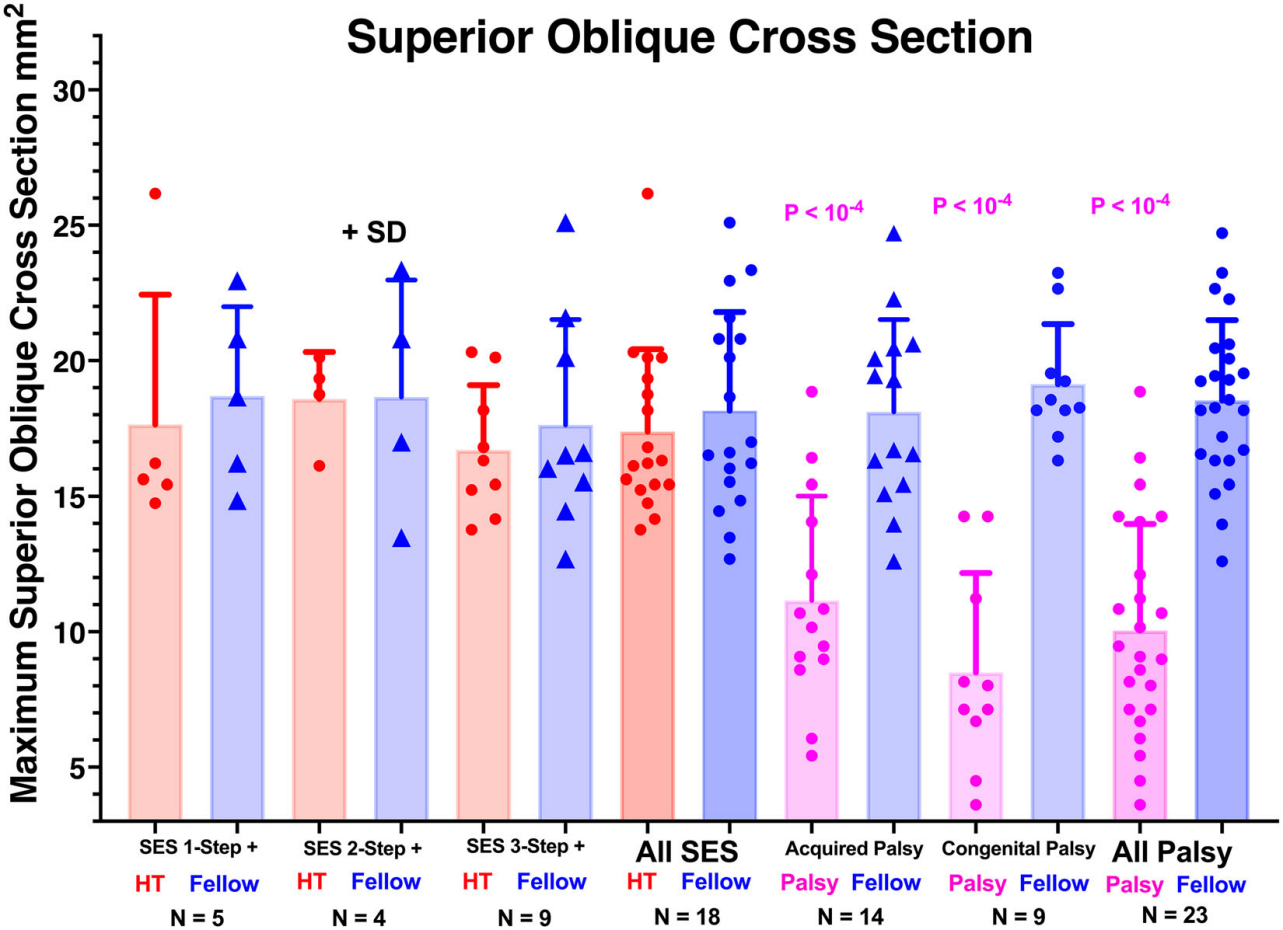
Maximum cross-sectional area of SO muscle in the HT and fellow orbits of patients with SES and acquired and congenital SOP. Symbols represent every individual muscle. By one-way ANOVA, all mean palsy cross-sections were highly significantly less than fellow cross-sections (*P* < 10^−4^) but not different from one another. None of the other non-palsied mean cross-sections differed from one another (*P* > 0.8).

Maximum SO cross-section was statistically indistinguishable at 16 to 18 mm^2^ in both the hypertropic and hypotropic orbits of subjects with SES, regardless of the number of 3ST steps fulfilled (*P* > 0.8 by one-way ANOVA) (Fig. 3). Maximum SO cross-section in the hypotropic fellow eye in SOP was also statistically indistinguishable from both SO muscles in SES (*P* > 0.8), but maximum cross-section of the SO with both congenital and acquired palsy was much smaller at 8–11 mm^2^ than either its fellow or the hypertropic or hypotropic SO in SES (*P* < 0.0001). These findings confirm that subjects categorized as having SES had normal SO size atypical of SOP, but that subjects having both congenital and acquired SOP exhibited similar unilateral SO atrophy indicative of SOP.

### Inferior Oblique Muscle

**Figure 4.**
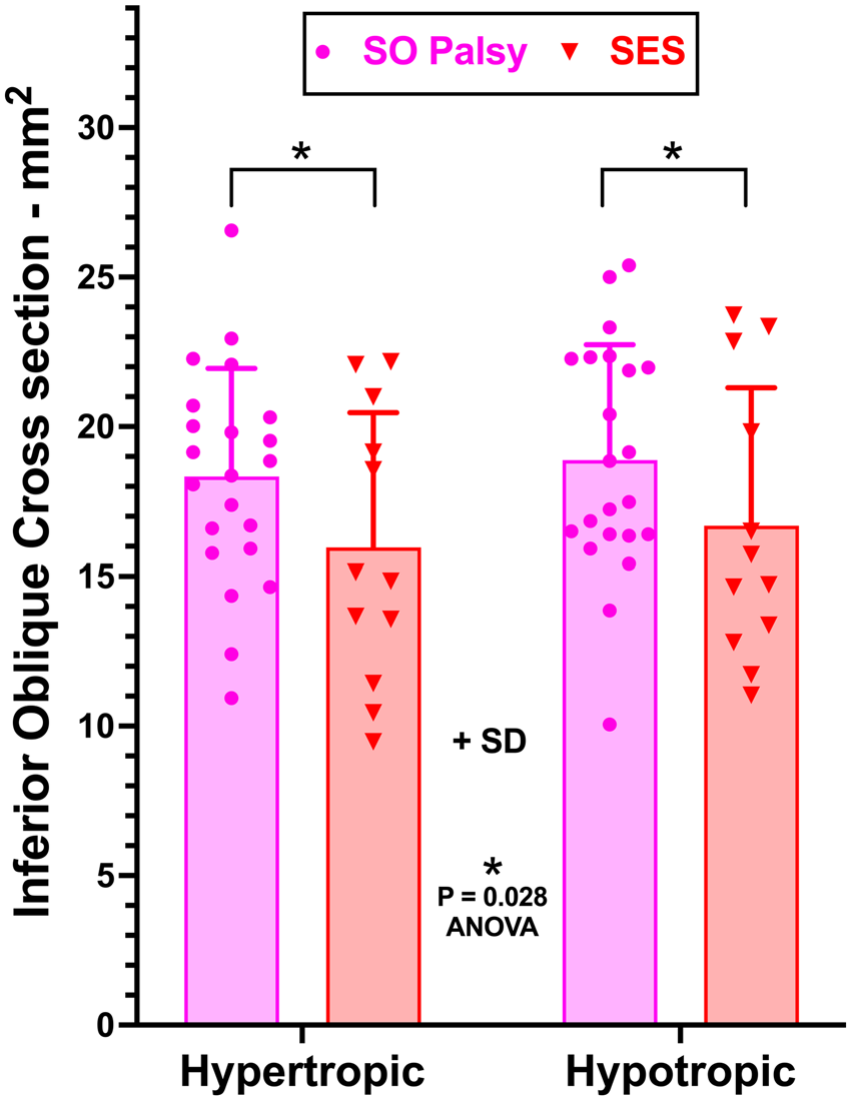
Mean IO muscle cross-section measured at the midpoint of the IR muscle in quasi-coronal MRI. Although two-way ANOVA demonstrated that the IO cross-section was significantly greater in SOP than in SES (asterisk), within each group the mean cross-sections in the hypertropic and hypotropic orbits did not differ significantly. Every datum is plotted for every subject as an individual symbol.

In contrast to the highly significantly smaller SO cross-section in SOP (Fig. 3), two-way ANOVA demonstrated no significant difference in IO cross-section between the hyper- and hypotropic orbits in either SOP or SES (Fig. 4). However, IO cross-section in both orbits in SOP averaged about 18 mm^2^, significantly greater by about 11% than the value of about 16 mm^2^ in SES (*P* = 0.028, two-way ANOVA).

### Rectus Pulley Locations

**Figure 5.**
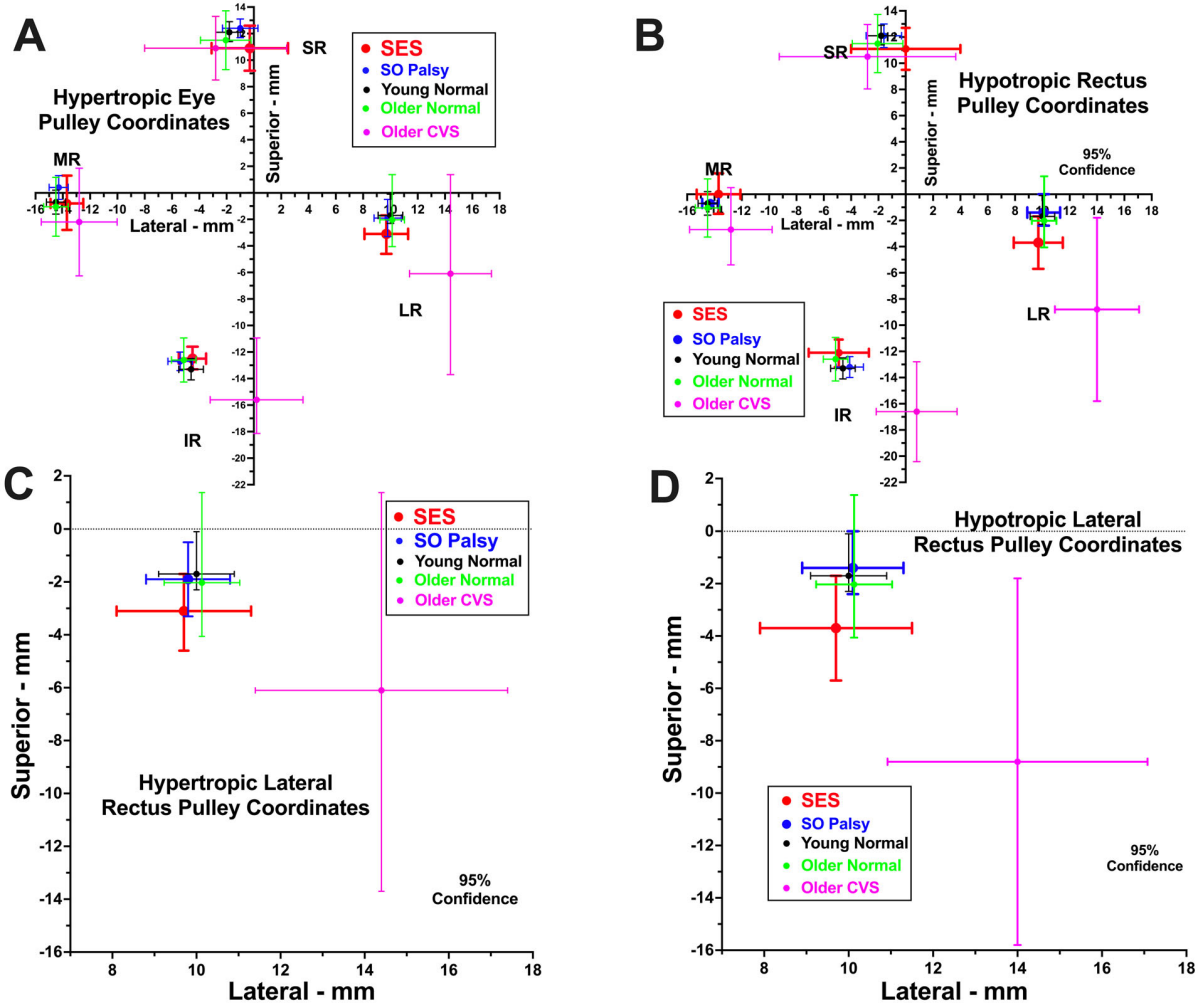
Rectus pulley positions in standardized oculocentric coordinate system for current subjects with SES who fulfilled the 3ST, as well as data published using identical technique in young normal subjects,^24^ older normal subjects,^24^ older subjects with CVS associated with SES,^24^ and subjects with SOP.^10^ (**A**) All rectus pulleys of hypertropic eyes. (**B**) All rectus pulleys of hypotropic eyes. (**C**) Expanded view of LR pulley of a hypertropic eye. (**D**) Expanded view of LR pulley of a hypotropic eye.

Rectus pulleys were located in the quasi-coronal plane for each orbit of subjects with SES who fulfilled the entire 3ST and are plotted in an oculocentric coordinate system in Figure 5 for comparison with published data obtained with identical technique in young normal subjects,^24^ older normal subjects,^24^ subjects with cyclovertical strabismus (CVS) associated with SES,^24^ and subjects with SOP.^10^ Figures 5A and 5B display the positions of all four rectus pulleys; it is evident that IR and LR pulley positions in elderly patients with CVS are much more certrifugally placed relative to globe center in both hyper- and hypotropic orbits than is the case for the current subjects with SES who fulfill the 3ST or SOP, or either control group. Because it is postulated that asymmetrical sag of the LR pulley causes hypotropia in SES, Figures 5C and 5D display in greater detail the positions of the hypertropic (Fig. 5C) and hypotropic (Fig. 5D) LR pulleys. Qualitatively consistent with expectation in cases of SES fulfilling the 3ST, the mean position of the hypotropic LR was lower at 3.7 ± 1.0 mm inferior to globe center, whereas that of the hypertropic LR was 3.1 ± 0.7 mm inferior (*P* = 0.075, paired two-tailed *t*-test) for a mean difference of 0.6 ± 0.8 mm. Although this difference was not significant at the 0.05 level, the differences in LR versus medial rectus (MR) vertical positions were significantly greater at −3.7 ± 1.0 mm in the hypotropic versus −2.4 ± 1.1 mm in the hypertropic orbit (*P* = 0.038, paired two-tailed *t*-test). The comparable differences in LR versus MR vertical positions reported by Chaudhuri and Demer^24^ for CVS were −6.1 mm for the hypotropic orbit and −3.9 mm for the hypertropic.

### Prism-Cover Hypertropia in Central Gaze

**Figure 6.**
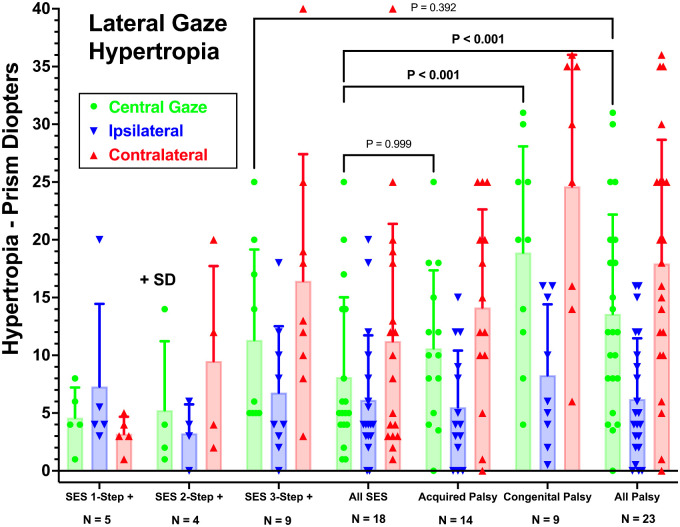
HT measured with prism-cover testing for a distant target in with head upright, contralesional lateral gaze, and ipsilesional lateral gaze for patients with SES and acquired and congenital SOP. Subjects with SES are subdivided according to the number of steps fulfilled in the 3ST and are combined as “All SES.” Acquired and congenital SOP are combined as “All Palsy.” Two-way ANOVA; note that HT values are statistically indistinguishable between SES fulfilling all parts of the 3ST and all SOP (*P* = 0.392). Also statistically similar are all SES and acquired SOP. Because of the larger HT in congenital SOP, HT in the pooled group with SOP is significantly greater than in the pooled group with SES.

The first step in the 3ST is demonstration of ipsilesional HT in central gaze, which averaged 18.2 ± 9.5∆ in congenital SOP, 10.6 ± 6.7∆ in acquired SOP, and 13.6 ± 8.6∆ when these two groups were pooled (Fig. 6). In SES, HT averaged 8.1 ± 6.9∆. By two-way ANOVA, HT was statistically indistinguishable between cases of SES fulfilling all parts of the 3ST and the pooled group with SOP (*P* = 0.392). Also statistically similar was HT in all SES versus acquired SOP (*P* = 0.999). Because of the larger HT in congenital SOP, HT in the congenital and the pooled group with SOP was significantly greater than the pooled group with SES (*P* < 0.001, two-way ANOVA). One case of SOP was orthotropic in central gaze. Figure 6 subdivides the data according to the number of steps positive for SES and subdivides acquired from congenital SOP.

### Lateral Gaze Incomitance

A positive second step in the 3ST requires that the HT be greater in contralesional than ipsilesional lateral gaze. As evident from Figure 6, that condition on average was fulfilled in four of nine cases (44%) of SES in which the head tilt criterion of the 3ST was not fulfilled; in these cases, HT increased from 3.3 ± 2.5∆ in the ipsilateral version to 9.5 ± 8.2∆ in the contralateral version (Fig. 6). In pooled SOP, by contrast, mean HT increased from 6.2 ± 5.3∆ in the ipsilateral version to 18.0 ± 10.7∆ in the contralateral version (Fig. 6). Only one case of acquired SOP had 15∆ horizontally concomitant HT, whereas in another HT was greater in ipsilesional than contralesional lateral gaze. In the other 21 cases of SOP (91%), HT was greater in contralesional than ipsilesional lateral gaze, consistent with the 3ST. In all but one SOP case, HT was greater in the ipsilateral than contralateral version, but in one of these the difference was only 1∆ and in another only 2∆; the maximum ipsilesional SO cross-section was profoundly reduced in both of these cases.

### Head-Tilt Testing

**Figure 7.**
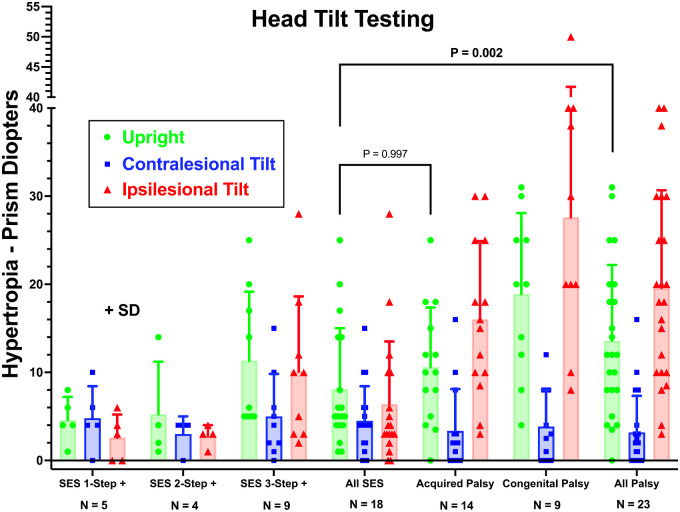
HT measured with prism-cover testing for a distant target in with head upright, contralesional head tilt, and ipsilesional head tilt for subjects with SES and acquired and congenital SOP. Subjects with SES are subdivided according to the number of steps fulfilled in the 3ST and are combined as “All SES.” Acquired and congenital SOP are combined as “All Palsy.” Two-way ANOVA; note that HT values are statistically indistinguishable between SES fulfilling all parts of the 3ST and acquired SOP (*P* = 0.997), although they are significantly different from the pooled group of all palsy cases due to larger values in congenital palsy.

**Figure 8.**
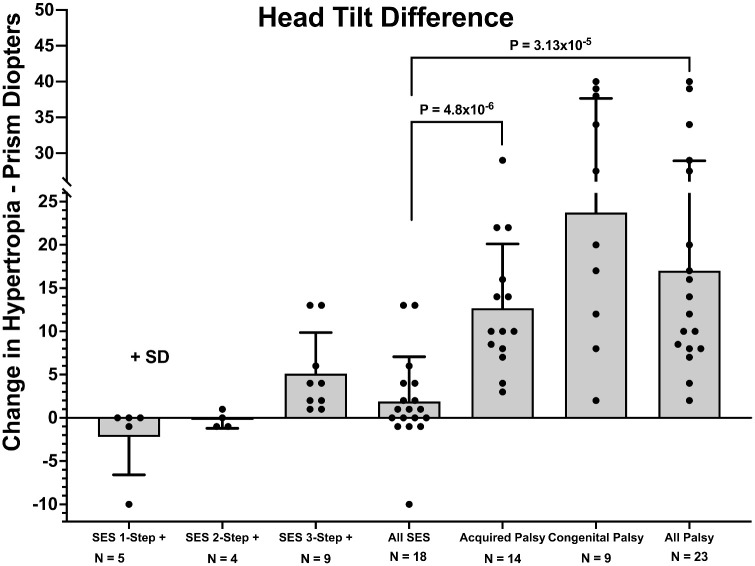
Increase in HT measured with prism-cover testing for a distant target from contralesional to ipsilesional head tilt for patients with SES and acquired and congenital SOP. Subjects with SES are subdivided according to number of steps fulfilled in the 3ST and are combined as “All SES.” Acquired and congenital SOP are combined as “All Palsy.” Note that the changes in HT values in both acquired and congenital SOP were significantly greater than in cases of SES in which the entire 3ST was fulfilled (two-tailed *t*-tests).

In every case of SOP, HT varied with head tilt in conformity with the 3ST, averaging (over all cases) 19.6 ± 11.1∆ in ipsilateral and 3.2 ± 4.1∆ in contralateral tilt (Fig. 7). In nine of 18 cases of SES (50%), the complete 3ST was fulfilled, so that the average HT in ipsilateral tilt of 10.1 ± 8.5∆ was greater than 5.0 ± 4.8∆ in the contralateral tilt. Note that HT magnitude in each head position was statistically no different in SES that fulfilled the complete 3ST versus acquired SOP (Fig. 7). However, change in HT between ipsilateral and contralateral tilts was less at 5.1 ± 4.8∆ in SES fulfilling the complete 3ST than the 13.1 ± 7.5∆ in acquired (*P* = 0.011) and 22.2 ± 13.7∆ in congenital SOP (*P* = 0.003) (Fig. 8).

### Torsion

Excyclotropia measured using double Maddox rods averaged 8.3 ± 4.9° in SES and was not significantly different from 6.0 ± 4.9° in SOP (*P* = 0.44, two-tailed *t*-test).

### Hess Screen Testing

**Figure 9.**
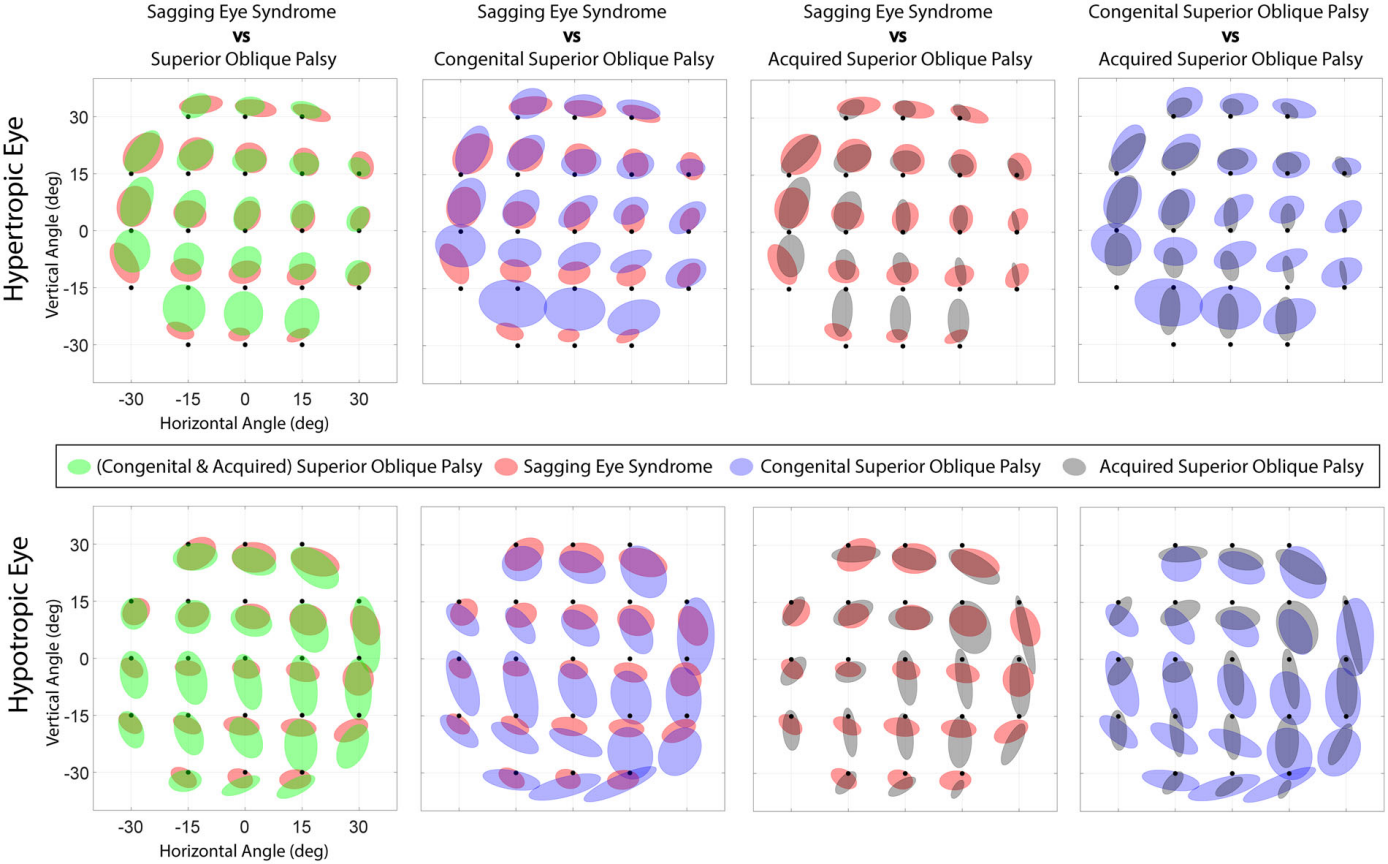
The 50% bivariate confidence regions of Hess screen alignment for subjects with SES and SOP. (*Top*) Positions of the hypertropic eye during hypotropic eye fixation. (*Bottom*) Positions of the hypotropic eye during hypertropic eye fixation. Data for right hypertropia are digitally reflected to correspond to left hypertropia for graphical purposes.

Hess screen alignment data are summarized in Figure 9, which plots the elliptical 50% confidence regions plotted about the mean alignment at each of the 21 tested gaze positions for the hypertropic eye with the fellow fixing (Fig. 9, top) and each of 21 gaze positions for the hypotropic eye with the fellow fixing (Fig. 9, bottom). The 50% confidence regions were chosen to provide an indication of data variability while maintaining graphical clarity, as larger regions, such as 95%, would have caused excessive graphical overlap among adjacent fixation positions. The data are plotted in this summary as if for left HT, after mirror reflection for cases of right HT. The bivariate confidence ellipses are each centered on the mean deviations for each target fixation point and are represented in green for pooled SOP and in red for SES. It should be noted that these confidence intervals encompass only half of the data points, with the remainder dispersed outside; this depiction avoids overlapping scatter of outlying data that would be confused with those for nearby fixation targets. As evident from Figure 9, the confidence ellipses for the two conditions nevertheless overlap at nearly every fixation point, although the average values differ in infraduction and adduction of the hypertropic eye (Fig. 9, top) and infraduction and abduction of the hypotropic eye (Fig. 9, bottom).

**Figure 10.**
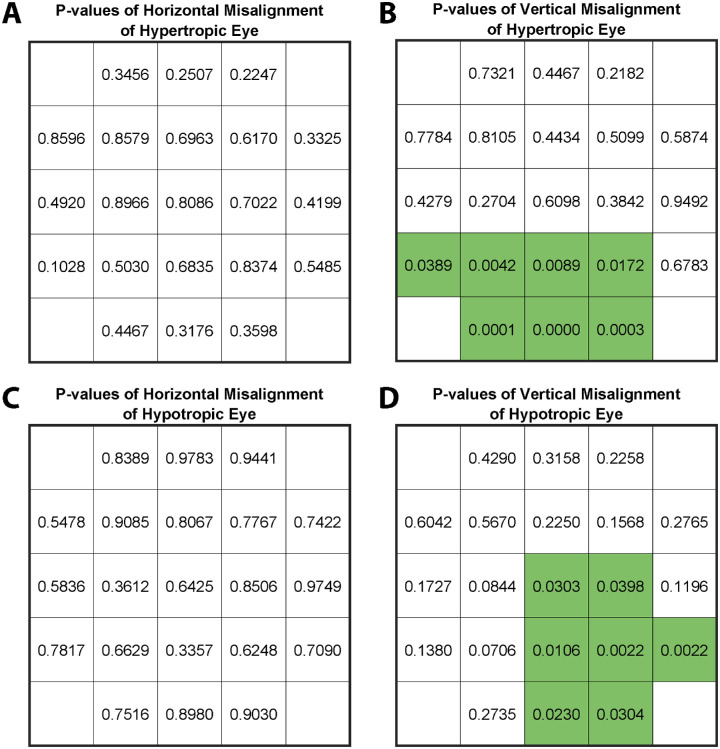
Statistical significance by two-tailed *t*-tests of differences in horizontal and vertical alignment comparing SOP and SES groups at gaze positions corresponding to the Hess screen alignment data in Figure 8. *P* values are separately plotted for horizontal and vertical deviations at these target positions, with values for *P* < 0.05 highlighted in *green*. Note that the two groups do not significantly differ in horizontal alignment at any of the 42 fixation conditions. Vertical alignment differences are significant for 14 central or inferior fixation conditions (two-tailed *t*-tests).

The statistical significances of differences in mean horizontal and vertical alignment measured by Hess screen testing are shown in Figures 10A and 10B for the hypertropic eye during fixation by the hypotropic eye, and in Figures 10C and 10D during fixation by the hypotropic eye during fixation by the hypertropic eye. As in Figure 9, the data are digitally reflected as necessary to appear as left hypertropia. Probabilities of significant differences in horizontal deviations are represented in Figures 10A and 10C and those for vertical deviations in Figures 10B and 10D. Although the two groups show no statistically significant differences in horizontal alignment, vertical alignment differences were significant for multiple inferior target positions with either eye fixating.

### Analysis by Machine Learning

We first tested whether ML can distinguish SES from SOP using Hess screen measurements. We found that use of all 84 HST measures actually degraded ML performance compared with the use of only the 14 target positions that differed statistically between the SES and SOP groups (*P* ≤ 0.05 in Figs. 10B and 10D). Therefore, due to the limited number of patient cases, we present ML results using 14 target positions, not 84 positions. As summarized in the Table, ML usually classified correctly. Overall accuracy, defined as the number of correctly classified patients divided by the total patients, varied among classifiers, ranging from 75.6% to 87.8%. The area under the receiver operating curve (AUC) is equivalent to the probability of correct classification in a two-alternative, forced-choice decision.^50^ A maximum AUC of 0.87 was achieved by the support vector machine method, and maximum overall accuracy (88%) was achieved by the K nearest-neighbor method.

**Table.** Summary of Classification Performance by Five Machine Learning Methods

Measures Incorporated	Decision Tree	KNN	SVM	Naïve Bayes	Ensemble
14 HST measures^*^					
Overall accuracy	78%	88%	83%	76%	83%
AUC	0.75	0.79	0.87	0.83	0.80
Nine measures from prism-cover test and torsion testing^†^					
Overall accuracy	78%	85%	85%	78%	90%
AUC	0.81	0.93	0.91	0.85	0.93
14 HST measures + nine measures from prism-cover test and torsion testing^‡^					
Overall accuracy	76%	83%	80%	78%	88%
AUC	0.75	0.84	0.65	0.74	0.91

The same measurement group was employed in each case for training and testing. KNN, K-nearest neighbor method; SVM, support vector machine method.

* Alignment measures in only those fixation positions by Hess screen testing that differed statistically between SOP and SES groups.

† All prism-cover test and torsion measurements.

‡ Combination of HST and all prism-cover test and torsion testing.

We then investigated whether ML can distinguish SES from SOP by using eight prism-cover alignment measurements (central gaze HT, ipsilesional HT, contralesional HT, HT in infraversion, HT in sursumversion, HT in ipsilesional tilt, HT in contralesional tilt, and the difference in HT between ipsilesional and contralesional tilts), along with torsion measured by double Maddox rods. The results are provided in the Table. These nine alignment measurements enabled higher accuracy and AUC than those obtained from classifying Hess screening test data (Table). All classifiers achieved overall accuracy exceeding 78%, some as much as 90%; for all classifiers, AUC exceeded 0.81 and was as much as 0.93. These results imply that combining these commonly acquired alignment data using ML may produce more accurate diagnoses than using the standard 3ST. Even with this ML, however, diagnosis is imperfect.

Classification using the combined data for the nine non-Hess screen test alignment measures and 14 Hess screen test measures did not provide better diagnostic accuracy than only nine measurements (Table). The results imply that Hess screen test data do not improve on diagnostic accuracy obtainable from commonly acquired prism-cover, head tilt, and torsion data when used in ML to distinguish unilateral SOP from SES. However, Hess screen test data may still be useful to delineate comprehensive patterns of strabismus generally.

## Discussion

This study demonstrated that subjects with SES without SO atrophy can mimic all of the binocular alignment features of unilateral SOP. Fully half of all cases of SES fulfilled the classic 3ST test for SOP, although not all cases of actual SOP did so. Additional cases of SES fulfilled two of the steps of the 3ST. Although the average HT for SOP was larger and more dependent on head tilt than the average for SES, individual measurements in both groups varied widely and overlapped considerably. This was the case for both congenital and acquired SOP. Consistent with expectation, cases of SES that fulfilled the 3ST test had more inferior LR relative to MR pulley positions in the hypotropic than hypertropic orbit, although this difference was modest and smaller than earlier reported for cyclovertical strabismus associated with SES.

When alignment measurements of subjects with HT due to SES and SOP were compared, significant differences in vertical alignment were evident in downward gaze positions that include the expected field of action of the SO muscle. The average magnitude of HT tends to be greater in congenital SOP than in SES (Fig. 6). Demonstration of these group mean differences confirms that SO function does contribute to horizontal and vertical binocular alignment as classically expected, but the differences between SOP and SES are small relative to individual variability among patients. Moreover, subjective torsional misalignment demonstrated by double Maddox rod testing demonstrated no significant differences between SOP and SES, being nearly equal in the two.

Taken together, these findings indicate that conventional interpretation of clinical alignment measurements cannot distinguish unilateral SOP from SES in cases of HT. Can ML improve interpretation of alignment measurements sufficiently to distinguish the two diagnoses with certainty? The answer is that ML is better than interpretation of individual clinical measurements but nevertheless is imperfect. Automated interpretation using 14 target positions in which average alignment differed significantly between the groups yielded overall accuracy of 75% to 83% (Table). ML diagnoses using four individual approaches based on prism-cover testing data including HT and torsion yielded an overall accuracy of 78% to 85%, increasing to about 90% when all were combined as an ensemble (Table). There was no further improvement in overall accuracy when prism-cover and torsion measurements were combined with Hess screen test data. Note that these accuracy assessments were obtained from cross-validation, which reports the averaged predictive performance of a model on data independent from training data. Even with data from additional cases, perfect distinction between SOP and SES using ML is probably impossible because of the high degree of overlap in alignment patterns between SOP and SES (Fig. 8). It goes without saying that human differential diagnosis based on interpretation of clinical cases could not exceed the ideal machine performance of 80% to 90% and would likely be worse.

When, then, might the 3ST fail to distinguish SOP from HT associated with SES? The likely explanation is that neither SO weakness per se nor rectus pulley sag alone constitutes sufficient cause for the large angles of HT typically observed in such cases, especially the powerful effect of head tilt on the magnitude of hypertropia. Two classical computational modeling studies of extraocular muscle action demonstrate that the relatively large HT angles observed in SOP cannot be due to a deficiency of SO function alone but must also be augmented by secondary factors such as changes in activity of multiple other extraocular muscles.^14^^,^^15^ Some of the predicted changes have been confirmed by MRI,^43^ although there is no relationship between SO size and magnitude of the head tilt phenomenon in SOP.^19^ Our own preliminary modeling that includes modern concepts of rectus pulleys and compartmentalization also predicts only modest HT due to SO weakness alone or to asymmetrical LR pulley position alone (Q. Wei, unpublished data). It seems likely that the influence of head tilting on HT is a feature of neural adaptation to chronic HT generally rather than being caused by SO dysfunction specifically.

Bowing of the LR–SR ligament^51^ and sag^24^ of the LR more than MR muscles is very common in adults over age 50 years, yet only a minority of them develop strabismus. Cyclovertical strabismus has been reported in older subjects who have larger and asymmetrical sag of the LR pulley in the hypotropic orbit.^24^ We recently reported the existence of an entity termed “masquerading SOP,” which exhibits every feature described as characteristic of this entity but with normal SO size and contractility.^52^ Not only do cases of masquerading SOP have LR pulley positions statistically similar in the hyper- and hypotropic orbits that is inconsistent with SES as the cause of the HT, but also many masquerading cases are congenital or present much younger than possible for SES.^52^ The cause of masquerading SOP is unknown. Given these considerations, it is possible that the current cases of SES might have masquerading SOP and only incidentally have the relatively common findings of SES. Because masquerading SOP has no age limitations and an unknown etiology and lacks diagnostic anatomical features, this condition can never be excluded as a cause of HT that fulfills the 3ST.

An additional complexity is that not all cases of actual SOP are created equal. The SO muscle is selectively innervated in its medial and lateral compartments by separate trochlear branches^53^ that are probably differentially susceptible to denervation so as to create differing different patterns of atrophy and different patterns of vertical and torsional strabismus.^41^ Thus, it is not surprising that around 9% of cases with SO atrophy in the current study did not fulfill the 3ST.

Although this study involved a limited number of subjects with SOP and SES, it is a strength that all patients had prospectively acquired alignment data and were rigorously classified in objective fashion by MRI so as to eliminate diagnostic confusion as a potential confound. This provides confidence that ML algorithms were never trained with erroneously classified data. When it works, ML is robust when training includes a large dataset, and, to be persuasive, ML classification must be confirmed in a dataset different from the training set. The present dataset was relatively small, so if the ML approach used here had been able to distinguish SOP from SES, only a weak endorsement could have been drawn that would require confirmation in another dataset. However, none of the five ML approaches could perfectly distinguish SOP from SES. This supports the strong inference that, on the basis of alignment measurements, SOP cannot be clinically distinguished from SES in subjects with HT. Although that conclusion would not change even with a larger dataset, it should also be remembered that every clinical patient represents an individual dataset of only one. Although average alignment of groups of patients does differ for some gaze positions, alignment measurement cannot reliably distinguish SES from SOP for individual patients.

The current study relies on the assumption that SO muscle functional capability is reflected in its anatomical size. For atrophic SO muscles, it is highly plausible that contractile function is subnormal, as contractile change in SO cross-section from supraduction to infraduction varies correspondingly with SO cross-section itself.^33^^,^^54^^,^^55^ In subjects who have SES, could the converse be the case with weak SO muscles despite normal size? Although muscle size is largely preserved in the mitochondrial myopathy chronic progressive external ophthalmoplegia,^56^ this condition affects all extraocular muscles rather than the SO only and was excluded using clinical evidence from subjects in the current study. Myasthenia gravis can present with extraocular weakness without atrophy but was also excluded based on clinical features. In subjects who have SES, could SO tendons have dehisced to prevent transmission of SO force to the globe? Interruption of the SO tendon has been demonstrated to increase maximum SO cross-section as the muscle belly recoils posteriorly^57^; this increase was not observed here in cases of SES, so tendon defects are improbable. As demonstrated in Figure 3, IO size was similar in the hyper- and hypotropic eyes in both SOP and SES, making the IO unlikely to cause the HT in either condition. Although a heretofore unknown cause of SO weakness without atrophy can, of course, not be excluded, it would seem unlikely to explain the current findings

What should clinicians do in light of these findings? First, it would not be appropriate to presume the diagnosis of SOP in HT cases simply because the 3ST is positive. Such a presumptive diagnosis might likely be correct in a child or young adult in whom SES is highly improbable, but the presumption of SOP becomes progressively more likely be incorrect as patients exceed age 45 years. Indeed, SES causes more than half of all new diplopia in patients over age 90 years.^31^ A more descriptive diagnosis such as head-tilt–dependent HT would suffice and not exceed the evidentiary basis.
